# CD44 Marks Dormant Tumor Cells After HER2 Inhibition in Breast Cancer Cells

**DOI:** 10.3390/ijms26104907

**Published:** 2025-05-20

**Authors:** Carla Vargas, Adam Aguirre-Ducler, Karina Cereceda, Sebastián Quijada, Nicolás Escobar-Gómez, Rodrigo L. Castillo, Matías Escobar-Aguirre

**Affiliations:** 1 Departamento de Medicina Interna, Facultad de Medicina Oriente, Universidad de Chile, Santiago 8330014, Chile; carla.vargas.d@ug.uchile.cl (C.V.); rodrigouch@uchile.cl (R.L.C.); 2 Instituto Oncológico, Fundación Arturo López Pérez, Santiago 7500710, Chile; 3 Department of Pathology, Yale University School of Medicine, New Haven, CT 06520-8023, USA; adam.ducler@yale.edu; 4 Escuela de Tecnología Médica, Facultad de Ciencias de la Salud, Universidad San Sebastián, Santiago 7510157, Chile; squijadas1@correo.uss.cl (S.Q.); nescobarg1@correo.uss.cl (N.E.-G.)

**Keywords:** breast cancer, tumor dormancy, cancer stem-cells, CD44, tumor recurrence, genome-editing, JAK-STAT signaling

## Abstract

Therapy resistance remains a major barrier to improving outcomes in HER2-positive breast cancer, with dormant tumor cells (DTCs) and cancer stem cells (CSCs) playing critical roles in recurrence and treatment failure. Herein, we investigated the interplay between dormancy and CSCs features in HER2-amplified breast cancer cell models and evaluated the role of the JAK1–STAT3 axis in sustaining these therapy-resistant phenotypes. Using an in vitro dormancy model induced by HER2 inhibition, we observed a reversible quiescent state characterized by decreased proliferation and viability, accompanied by a significant increase in the CSC marker CD44. CD44 expression was rapidly induced following HER2 inhibition, preceding measurable effects on cell viability, and persisted throughout the dormancy phase. CD44-positive populations showed reduced sensitivity to HER2 inhibition and displayed robust proliferative recovery upon therapy withdrawal. Functional studies revealed that the inhibition of JAK1, but not STAT3, impaired the recovery of CD44-positive populations and decreased their proliferative capacity, suggesting a critical role for JAK1 in maintaining the CSC phenotype during therapy. These findings underscore the importance of CD44 as a marker and mediator of therapy resistance and suggest that targeting CD44-positive cells or the JAK1 signaling axis could improve the efficacy of HER2-targeted therapies. Our study provides novel insights into the mechanisms underlying dormancy and CSC induction in HER2-positive breast cancer and highlights potential strategies to mitigate therapy resistance and prevent disease recurrence.

## 1. Introduction

Breast cancer continues to be the foremost cause of cancer-related fatalities among women across the globe despite significant strides in early detection and treatment methodologies. The primary reason for this high mortality rate is the emergence of incurable recurrences that can occur many years or even decades after initial treatment targeting the primary tumor [1,2]. These recurrences often stem from residual tumor cells (RTCs) that evade therapy, typically existing in a dormant, non-proliferative state thought to be a form of reversible quiescence [3]. Targeting and depleting this pool of dormant RTCs by disrupting their survival mechanisms offer a promising strategy to mitigate the risk of breast cancer recurrence [4,5].

Dormant tumor cells reside as solitary cells or as micrometastases, which remain non-proliferative for long periods of time before reactivating [6,7]. The use of genetically engineered mouse models (GEMMs) of oncogene-driven tumor growth has enabled the identification of pathways underlying the dormant state [8,9,10]. Importantly, gene expression signatures from GEMMs applied to clinical specimens have found significant correlations that validate the use of experimental models to uncover the mechanisms of tumor dormancy in breast cancer [11,12,13,14,15].

Dormant cells display stem cell-like features that are influenced by the microenvironment. Cancer stem cells (CSCs) are a therapy-resistant subgroup with renewal capacity and mesenchymal features and often identified positive for Cluster of Differentiation-44 (CD44) [16,17,18]. CD44 is a surface glycoprotein that plays significant roles in various physiological processes, including cell adhesion, migration, and activation, as well as in maintaining normal stem cell functions. Its fundamental role as a receptor for hyaluronic acid (HA), a major component of the extracellular matrix, positions CD44 as an essential mediator in cellular interactions with the surrounding microenvironment [19]. CD44 is expressed in various cell types, including immune cells, fibroblasts, and epithelial cells, where it facilitates cell adhesion by binding to HA. This function is notably vital in hematopoiesis, where CD44 participates in stem cell maintenance and proliferation [20]. In the context of tumorigenesis, CD44 is increasingly recognized as an essential marker and functional component in breast cancer stem cells, playing a vital role in their maintenance, tumorigenesis, and malignancy. Evidence substantiates that CD44 not only serves as a cell surface marker for CSCs but is also involved in various pathways that significantly influence stemness features [21].

Several studies have shown that CD44^+^/CD24^−^ tumor cells represent a poor prognosis factor associated with more aggressive outcomes [22,23,24,25]. In breast cancer, CSCs can emerge after treatment of HER2-amplified tumors, a process mediated by IL-6 signaling that operates in an autocrine fashion to induce a CSC phenotype in response to HER2 inhibition [26], similar to the effect of notch signaling in survival and proliferation of CSCs [9,27]. Overall, stem cell and dormant features enable therapy resistance and survival to treatment in breast cancer cells. However, it remains unclear whether tumor cells with these features exist prior to therapy or are rather induced by the treatment. Additionally, to what extent are CSCs and dormant tumor biologically distinguishable and, therefore, respond to different stimuli and display distinct vulnerabilities to treatment?

In this work, we developed an in vitro assay to trigger dormancy in HER2-amplified human breast cancer cells and studied the induction of CSC features, as well as the function of JAK1 and STAT3, components of the IL-6–STAT3 axis. We inhibited HER2 activity using lapatinib, dual tyrosine kinase inhibitor targeting the ErbB1 (EGFR) and ErbB2 (HER2) receptors, that has been shown to effectively disrupt HER2 signaling, inhibiting cell proliferation and survival in breast cancer cells [28,29], and used primarily to treat HER2-positive breast cancer [30,31].

After HER2 inhibition, tumor cells acquire a reversible quiescence state, which can be observed shortly after therapy exposure. In this phase, the CSC marker CD44 is rapidly induced and maintained if dormancy persists. While JAK1 and STAT3 inhibition does not appear to influence CD44 induction, the JAK1 function appears to influence resistance to HER2 inhibition. Our study contributes to understanding the intricate mechanisms of therapy resistance that implicate dormancy and CSC induction in human breast cancer cells.

## 2. Results

### 2.1. HER2-Amplified Breast Cancer Cells Display Dormancy After Therapy

**Figure 1:**
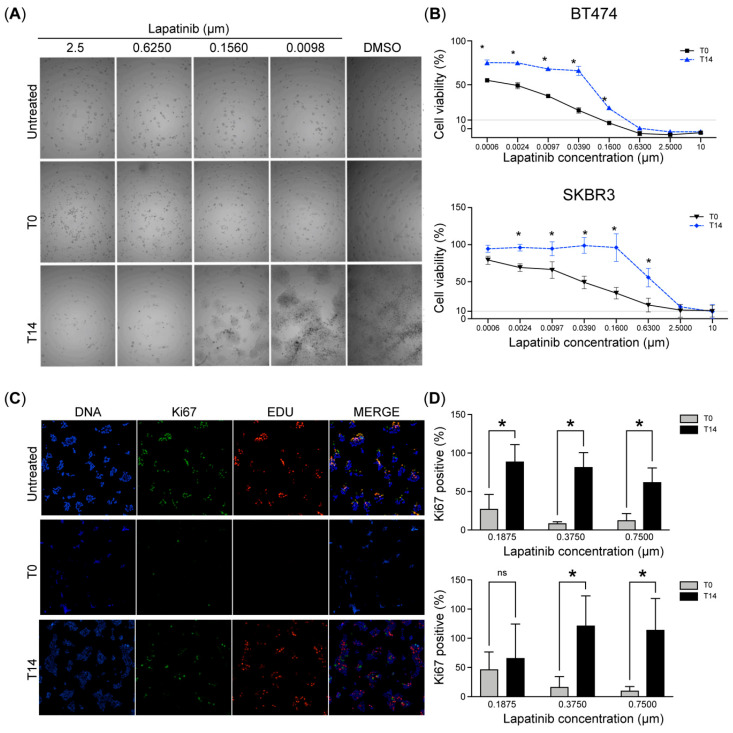
Dormancy induction after HER2 inhibition in human breast cancer cells. (**A**). Representative brightfield images of BT-474 cells treated with lapatinib at different concentrations for 4 days (T0) and recovery after 14 days of lapatinib removal (T14). (**B**). Dose–response curves of HER2-amplified breast cancer cell lines BT-474 and SK-BR-3 to lapatinib (0.0006, 0.0024, 0.0097, 0.039, 0.16, 0.63, 2.5, and 10 µM). Cell viability was assessed using an MTT assay. (**C**). Representative images of BT-474 immunofluorescence assays for Ki67 (green) and EDU (red) at basal (untreated), lapatinib treatment (T0), and recovery T14. (**D**). Bar charts illustrate the Ki-67-positive ratio of BT-474 (**upper**) and SK-BR-3 (**lower**) cells. Data were normalized to the control (vehicle) and presented as the mean ± SD. In vitro analyses are representative of at least three independent experiments. Kruskal–Wallis tests were used to assess differences between groups, and Dunn’s test was applied for post hoc analysis. * *p* < 0.05; ns, not significant.

To investigate dormancy induction in tumor cells, we developed an in vitro assay designed to pharmacologically induce a reversible, non-proliferative state in the HER2-amplified cell lines BT-474 and SK-BR-3. Viability was assessed following a four-day treatment with lapatinib (herein referred to as T0) across a concentration range of 0.006 to 10 µM. The results demonstrated a significant reduction in cell viability at all concentrations tested, with concentrations above 0.16 and 2.5 µM achieving over 90% inhibition in BT-474 and SK-BR-3 cells, respectively. Lapatinib exerted a more pronounced effect on BT-474 cells, reflected by an IC_50_ value of 0.031 µM (CI: 0.017–0.05 µM), compared to an IC_50_ of 0.08 µM (CI: 0.03–0.16 µM) in SK-BR-3 cells (Figure 1A,B). After the 4-day treatment, lapatinib was removed, and the cells were allowed to recover for 14 days (herein referred to as T14). During this recovery period, SK-BR-3 and BT-474 cells resumed proliferation significantly, except at concentrations of 0.63, 2.5, and 10 µM for BT-474 and 2.5 and 10 µM for SK-BR-3. These results suggest that our assay effectively induces a reversible dormancy state through HER2 inhibition.

Given that the hallmark of dormant tumor cells is a reversible, non-proliferative (quiescent) state, we used Ki67 as a marker to assess proliferative activity during treatment. Lapatinib treatment at 0.1875, 0.375, and 0.75 µM in BT-474 cells resulted in mean Ki67 positivity rates of 27%, 8%, and 12%, respectively, and 46%, 17%, and 10% in SK-BR-3 cells. Notably, at T14, Ki67-positive cells increased significantly from T0, reaching 88%, 81%, and 61% in BT474 and 65%, 120%, and 112% in SKBR3 at these respective concentrations (Figure 1C,D). We also assessed proliferation using EdU incorporation, a marker of S-phase entry. At T0, EdU-positive cells were nearly undetectable after HER2 inhibition, supporting the complete proliferation arrest during treatment (Figure 1C). After recovery upon drug withdrawal, EdU-positive cells were re-established similar to basal levels. These findings indicate that potent HER2 inhibition induces a reversible, non-proliferative state consistent with dormancy, as demonstrated by Ki67- and EdU-negative SK-BR-3 and BT-474 cells during treatment that subsequently regained proliferative capacity upon therapy withdrawal.

### 2.2. Dormant Cells Are Predominantly CD24-Negative and CD44-Positive

**Figure 2:**
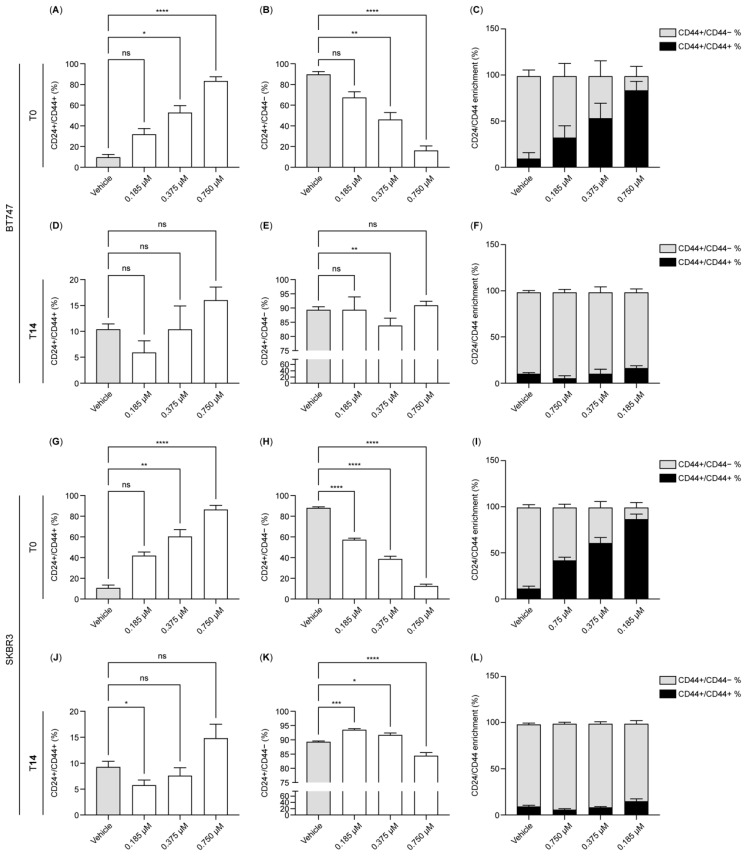
HER2 inhibition induces CD44 in dormant breast cancer tumor cells. (**A**–**L**). The bar charts illustrate the expression of CD24 and CD44 in BT-474 (**A**–**F**) and SK-BR-3 (**G**–**L**) measured by flow cytometry at T0 (**A**–**C**,**G**–**I**) and T14 (**D**–**F**,**J**–**L**) using lapatinib concentrations of 0.1875, 0.375, and 0.75 µM. Data were normalized to the control (vehicle). Data are the mean ± SD. * *p* < 0.05, ** *p* < 0.01, *** *p* < 0.001, **** *p* < 0.0001. T0: 4 days after lapatinib treatment; T14: 14 days after drug removal; DMSO: dimethyl sulfoxide (vehicle control for lapatinib).

We investigated the dynamics of the cancer stem cell marker CD44 during dormancy induction and exit (Figure 2A–L). In the untreated state, BT-474 and SK-BR-3 cells—characterized by their epithelial origin—were predominantly CD24^+^/CD44^−^, constituting approximately 90% of the population (vehicle control; Figure 2B,H). In contrast, the CD24^+^/CD44^+^ double-positive population comprised less than 10% in both cell lines (Figure 2A,G). Upon HER2 inhibition, we observed a marked shift: the CD24^+^/CD44^+^ population rose to approximately 90% with the 0.75 µM treatment (Figure 2A,G), with statistically significant increases at concentrations of both 0.375 and 0.75 µM in BT-474 and SK-BR-3 cells. Conversely, the CD24^+^/CD44^−^ population was significantly reduced under HER2 inhibition (Figure 2B–C,H–I).

Interestingly, at T14, the CD24^+^/CD44^−^ population was restored to around 90% positivity at 0.375 and 0.75 µM, except in the 0.1875 µM condition in SK-BR-3 cells (Figure 2E,K). The double-positive CD24^+^/CD44^+^ population, although fluctuating with different treatments, remained below 20% and showed no statistically significant differences from control levels, except at 0.1875 µM in SK-BR-3 cells (Figure 2D,J). These findings suggest that cells surviving HER2 inhibition in a dormant state predominantly express the cancer stem cell marker CD44, with the CD24^+^/CD44^+^ population prevailing under strong HER2 inhibition. This alteration is reversible, as oncogene reactivation led to a predominance of the CD24^+^ population in both BT-474 and SK-BR-3 cells.

### 2.3. Early CD44 Induction Precedes Loss of Viability After HER2 Inhibition

**Figure 3:**
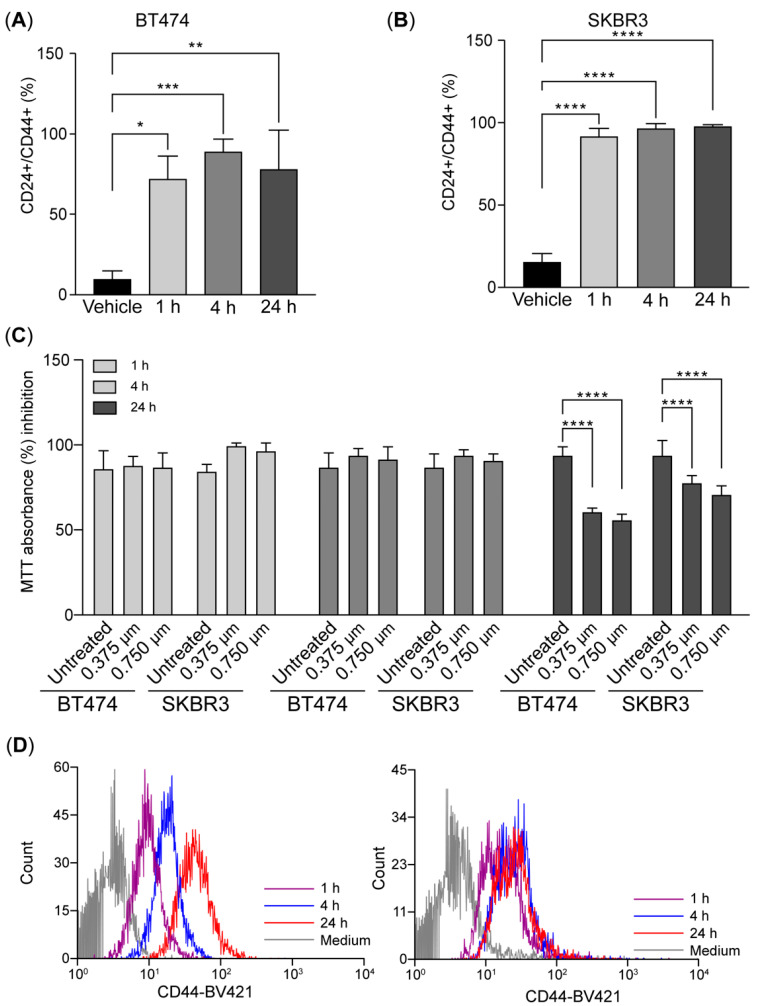
CD44 is induced shortly after HER2 inhibition in breast cancer tumor cells (**A**–**D**). The bar charts illustrate the expression of CD24 and CD44 in BT-474 (**A**) and SKBR3 (**B**) measured by flow cytometry at 1, 4, and 24 h after treatment with 0.75 µM of lapatinib. (**C**). Histograms of CD44 expression after treatment. (**D**). Cell viability was assessed using an MTT assay in BT-474 and SK-BR-3 cells. Data were normalized to the control (vehicle). Data are the mean ± SD. * *p* < 0.05, ** *p* < 0.01, *** *p* < 0.001, and **** *p* < 0.0001. T0: 4 days after lapatinib treatment; T14: 14 days after drug removal; DMSO: dimethyl sulfoxide (vehicle control for lapatinib).

We evaluated the onset of CD44 induction after HER2 inhibition. We measured the induction after 1, 4, and 24 h of treatment. Surprisingly, the CD24^+^/CD44^+^ population increased ~6 fold after 1 h treatment, and this was maintained at 24 h of treatment (Figure 3A–C). CD44 induction correlated with a decrease in cell viability as a result of HER2 inhibition that was only visible after 24 h of treatment in BT-474 and SK-BR-3 cells (Figure 3C). This result suggests a rapid induction of CD44 preceding the effect of HER2 inhibition on cell viability.

#### CD44-Expressing Tumor Cells Exhibit Sustained Proliferative Potential Following HER2 Inhibition

**Figure 4:**
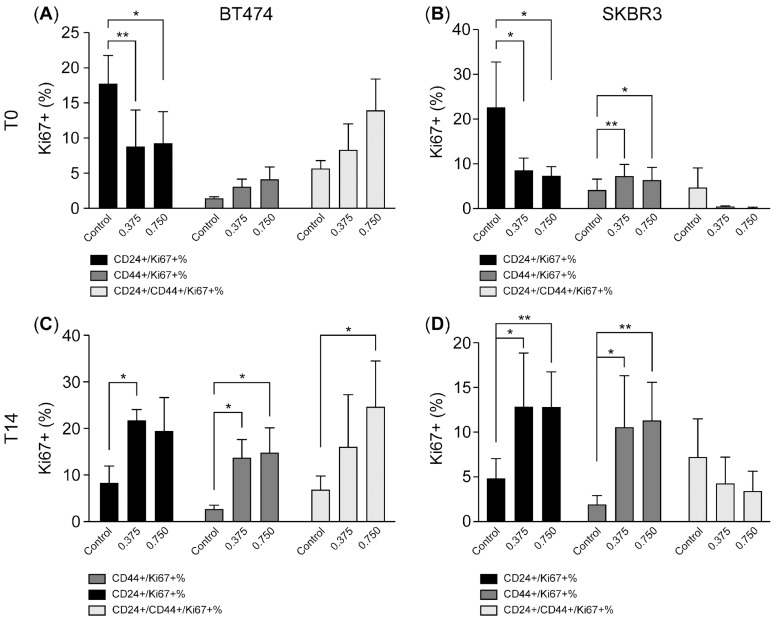
CD44-expressing tumor cells exhibit sustained proliferative potential following HER2 inhibition. (**A**–**D**). The bar charts illustrate the expression of CD24 and CD44 in Ki67-positive BT-474 (**A**,**C**) and SK-BR-3 (**B**,**D**) subpopulations measured by flow cytometry at T0 and T14 after treatment with 0.375 and 0.75 µM of lapatinib. Data are the mean ± SD. * *p* < 0.05, ** *p* < 0.01. T0: 4 days after lapatinib treatment; T14: 14 days after drug removal; DMSO: dimethyl sulfoxide (vehicle control for lapatinib).

To further characterize the proliferation status of CD44-expressing tumor cells during and after HER2 inhibition, we analyzed Ki67 positivity within CD24^+^ and CD44^+^ subpopulations (Figure 4A–D). In BT-474 cells, HER2 inhibition led to a significant reduction in the Ki67-positive CD24^+^ population at T0 (Figure 4A), indicating strong suppression of proliferative activity in these cells. In contrast, CD44^+^/CD24^−^ and CD24^+^/CD44^+^ populations exhibited much lower baseline Ki67 positivity and showed minimal additional reduction after HER2 inhibition, suggesting that these populations were inherently less proliferative and more resistant to therapy-induced proliferation arrest (Figure 4C).

A similar pattern was observed in SK-BR-3 cells. Lapatinib treatment significantly decreased Ki67 positivity within the CD24^+^ population (Figure 4B), while the Ki67-positive fraction among CD44^+^/CD24^−^ and CD24^+^/CD44^+^ populations remained relatively low and unchanged (Figure 4D). These findings indicate that HER2 inhibition preferentially suppresses proliferation in CD24^+^ tumor cells, while CD44-expressing populations are comparatively unchanged.

After drug withdrawal and during the recovery phase, both cell lines demonstrated a resumption of proliferation, though the dynamics differed between subpopulations. In BT-474 cells, Ki67 positivity in the CD24^+^ population returned to near-basal levels, while Ki67-positive CD44^+^/CD24^−^ and CD24^+^/CD44^+^ cells expanded significantly compared to T0, indicating robust proliferative recovery of CD44-expressing populations (Figure 4C). Similarly, in SK-BR-3 cells, Ki67 positivity increased in both CD24^+^ and CD44^+^ subpopulations after recovery (Figure 4D), although CD44^+^ populations remained at overall lower absolute levels compared to BT474 cells.

Taken together, these data suggest that CD24^+^ tumor cells are more sensitive to HER2 inhibition-induced proliferation arrest, while CD44-expressing tumor cells display a survival advantage under therapeutic pressure. Importantly, CD44^+^ cells retain the ability to rapidly re-enter the cell cycle upon therapy withdrawal, supporting their potential role as dormant, therapy-resistant tumor cells capable of driving tumor recurrence.

### 2.4. Effect of Inhibiting the JAK1–STAT3 Axis in CD44-Positive Populations

Previous studies have demonstrated the role of IL-6 signaling in promoting CD44 expression and its importance in conferring cancer stem cell properties [23]. Given our observation that CD44-positive populations exhibit reduced sensitivity to HER2 inhibition, we hypothesized that these CD44-positive populations require the JAK1–STAT3 signaling axis—downstream of IL-6—to sustain their CD44 identity.

**Figure 5:**
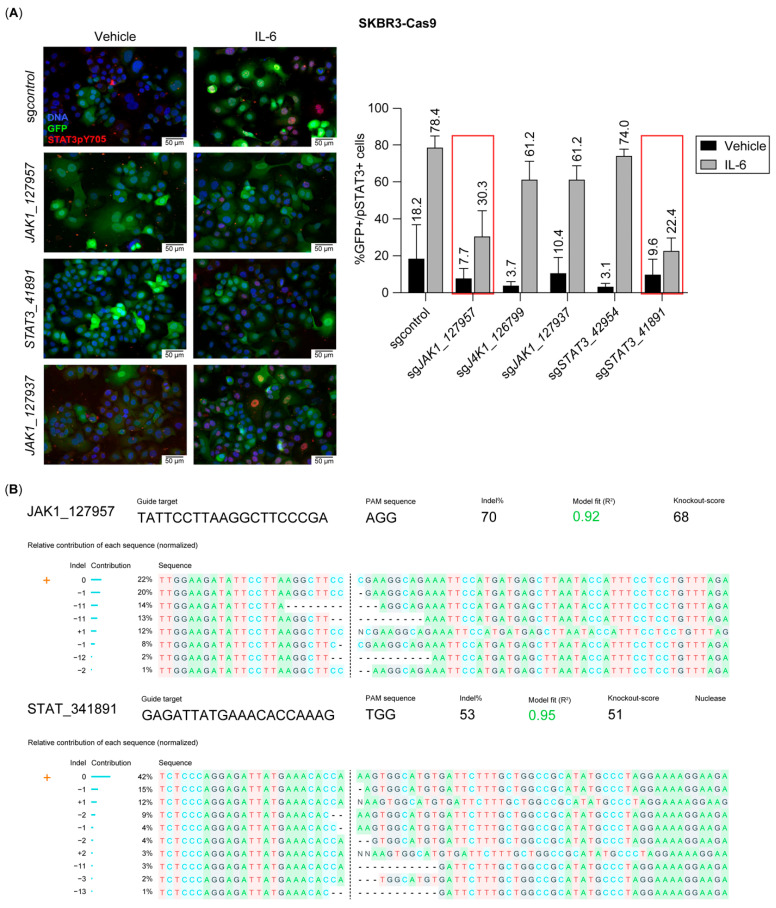
Selection of JAK1- and STAT3-directed sgRNAs showing blockade to an IL-6 stimulus. (**A**). Representative immunofluorescence images of SK-BR-3-Cas9 cells expressing sgRNAs targeting Jak1 or Stat3 genes, stained for STAT3pY705 after an IL-6 stimulus. GFP is expressed constitutively in SK-BR-3-Cas9 cells. Chart displays the quantification of STAT3pY705 as readout to select the best-performing sgRNAs for inhibiting IL-6 activity. Red boxes on sgRNAs show the highest effect. (**B**). Inference of CRISPR Edits (ICE) analysis for *sg*Jak1_127957 and *sg*Stat3_41891 displaying the proportion of indels in the population.

To test this hypothesis, we engineered SK-BR-3 cells to stably express Cas9 (SK-BR-3-Cas9) and subsequently introduced a lentiviral vector encoding GFP and sgRNAs targeting JAK1 and STAT3 genes. We included at least two sgRNAs per target gene, along with a non-targeting (control) sgRNA. To validate sgRNA efficacy, we stimulated SK-BR-3-Cas9 cells with IL-6 and quantified STAT3 phosphorylation as a readout using immunofluorescence staining to assess STAT3 activation (Figure 5A). Among the tested sgRNAs, JAK1-957 (sgJAK1) and STAT3-891 (sgSTAT3) demonstrated the highest inhibition of STAT3 phosphorylation relative to the control sgRNA. Additionally, PCR amplification of the loci flanking the sgRNA recognition sites followed by Inference of CRISPR Edits [32] (ICE) analysis confirmed that sgJAK1 and sgSTAT3 efficiently induced indels, predicted to result in loss-of-function mutations in a substantial fraction of the tumor cells (Figure 5B).

**Figure 6:**
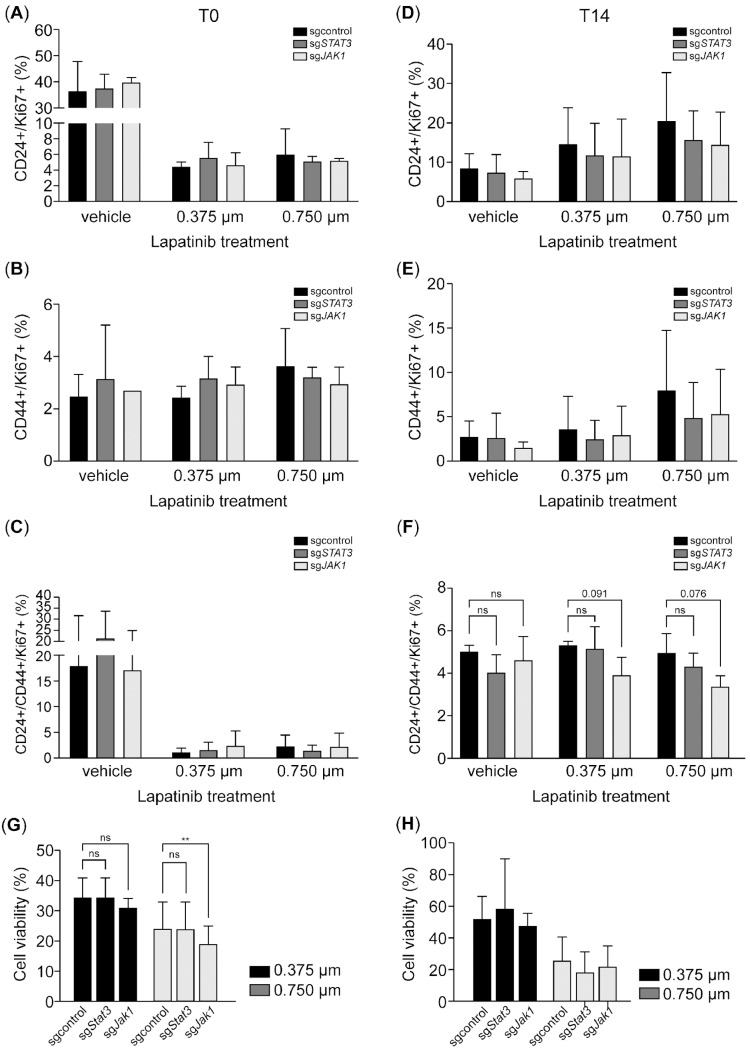
JAK1 inhibition impairs proliferation in the CD24^+^/CD44^+^ subpopulation during recovery after HER2 inhibition. (**A**–**F**). The bar charts illustrate the expression of CD24 and CD44 in Ki67-positive SK-BR-3-Cas9 cells expressing *sg*JAK1 or *sg*STAT3 after treatment with 0.375 and 0.75 µM of lapatinib at T0 (**A**–**C**) and T14 (**D**–**F**). (**G**,**H**). Treatment effect on cell viability using an MTT assay. Data are the mean ± SD. ** *p* < 0.01, ns = not significant. T0: 4 days after lapatinib treatment; T14: 14 days after lapatinib removal; DMSO: dimethyl sulfoxide (vehicle control for lapatinib).

**Figure 7:**
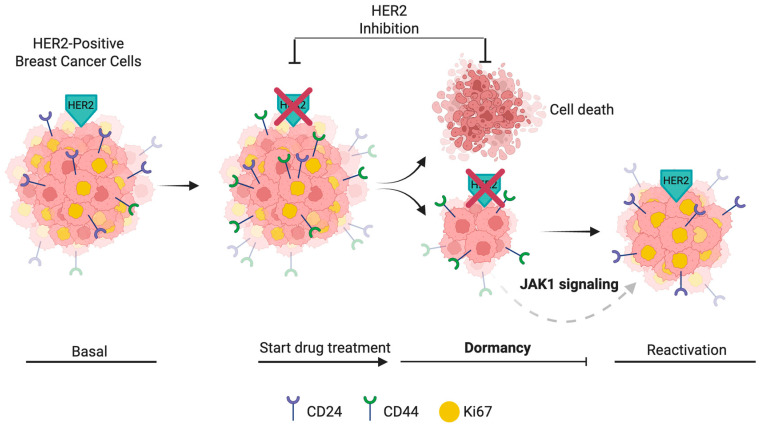
Model of dormancy induction and CD44 dynamics in HER2-positive breast cancer cells. HER2-positive breast cancer cells initially display a proliferative CD24^+^/CD44^−^ (Ki67^+^) phenotype (Basal). Upon HER2 inhibition, CD44 expression is rapidly induced. After extended HER2 inhibition (T0), most cells die, while a surviving population enters dormancy, characterized by CD44 expression and loss of Ki67. During the recovery phase (T14), reactivation occurs, with CD44^+^/Ki67^+^ cells contributing prominently to proliferation. CD24^+^ cells also reappear at this point. JAK1 signaling may support the maintenance and survival of dormant CD44^+^ populations during HER2 inhibition. Surface markers (CD24 and CD44) and nuclear Ki67 status are indicated for each phase. Created in BioRender. Vargas, C. (2025) https://BioRender.com/1loy830, accessed on 2 May 2025.

We next evaluated the impact of JAK1 and STAT3 inhibition on proliferation and CD44 induction following HER2 inhibition. While we observed trends in reduced Ki67^+^/CD24^+^ and Ki67^+^/CD44^+^ cell populations in response to JAK1 and STAT3 inhibition post-treatment, these differences did not reach statistical significance (Figure 6A–C). During the recovery phase, JAK1 inhibition further decreased the Ki67^+^/CD24^−^/CD44^+^ and Ki67^+^/CD24^+^/CD44^+^ populations, with the reduction in the Ki67^+^/CD24^+^/CD44^+^ group trending to statistical significance (*p* = 0.076) at the 0.75 µM lapatinib concentration (Figure 6D–F). Interestingly, JAK1 inhibition also reduced cell viability post-treatment; however, this effect was transient, with viability recovering by the T14 time point (Figure 6G–H). Overall, while STAT3 inhibition did not significantly impact cell viability, proliferation, or CD44 induction after HER2 inhibition, JAK1 inhibition appeared to sensitize cells to HER2 inhibition and impair proliferation during the recovery phase in CD24^+^/CD44^+^ tumor cells (Figure 7).

## 3. Methods

### 3.1. In Vitro Assays

BT-474 and SK-BR-3 cells were cultured in full medium DMEM/F-12 (Corning Life Sciences, 836 North St., Building 300, Suite 3401, Tewksbury, MA 01876, USA, Cat. # 10-103-CV) and DMEM High-glucose (Lonza, 90 Boroline Road, Allendale, NJ 07401, USA Cat. #12-604-F,), respectively, containing 10% fetal bovine serum (Merck, 400 Summit Drive, Burlington, MA 01803, USA Cat. # F0850), 1% penicillin/streptomycin (Thermo Fisher Scientific, 168 Third Avenue, Waltham, MA 02451, USA; Cat. # 15-140-122), and 1% glutamine (Thermo Fisher Scientific, Cat. # 25030081).

For in vitro dormancy experiments, the cells were first plated on full medium and, after 24–48 h (approximately 30% confluence), switched to medium containing 5% fetal bovine serum and lapatinib (Tocris Bioscience, 614 McKinley Place NE, Minneapolis, MN 55413, USA; Cat. # 6811) for four days of treatment, before being switched to 5% fetal bovine serum with no lapatinib for 14 days. Plates were harvested at treatment initiation, after treatment, and during the recovery phase as desired. The medium containing 5% fetal bovine serum and no lapatinib was replaced every 3 days.

MTT (Thermo Fisher Scientific, Cat. # M6494) assays were performed as per the manufacturer’s instructions. Briefly, reactive was added to a final concentration of 0.2 mg/mL and incubated for 2 h. Next, 10% SDS was used for solubilization, and absorbance was read at 570 nm.

### 3.2. Plasmids and Lentiviral Production

LentiV_Cas9_puro (Addgene, 490 Arsenal Way, Suite 100, Watertown, MA 02472, USA; Cat. # 108100) was used to generate Cas9-expressing SK-BR3 tumor cells. LRG2.1 (Addgene, Cat. # 108098) and LRG (Addgene, Cat. # 65656) vector backbones were utilized for cloning sgRNAs for CRISPR-Cas9 studies. For each sgRNA, sense and antisense oligos were phosphorylated, annealed, and then ligated into a BsmB1-digested vector. Ligated vectors were transformed into the chemically competent Stbl3 bacteria (Thermo Fisher Scientific, Cat. #C737303). Successfully transformed bacterial clones were picked from ampicillin-selective plates, and isolated DNA was sequenced using a U6 primer to confirm sgRNA incorporation.

Lentiviral particles were produced in HEK293T cells by transfecting 3 µg of pMD2.G (Addgene #12259), 6 µg of psPAX2 (Addgene #12260), and 9 µg of the sgRNA-expressing plasmid using polyethylenimine (PEI; Sigma-Aldrich, 3050 Spruce Street, St. Louis, MO 63103, USA; Cat. #919012) at a DNA:PEI ratio of 1:2. sgRNA lentiviruses were titered by serial dilution in SK-BR3 tumor cell lines using the fluorophore associated with the vector backbones as a readout on the Cytation5 (Agilent, Santa Clara, CA, USA).

### 3.3. Immunofluorescence

For in vitro immunofluorescence/labeling studies, SK-BR-3 and BT-474 tumor cells were cultured on glass coverslips (Bellco Glass Inc., 340 Eastdridge Avenue, Vineland, NJ 08360, USA; Cat. # 1943-010015A) and treated with 5 µM of EdU (Thermo Fisher Scientific, Cat. # C10640) for 4 h before harvest. EdU (5-ethynyl-2′-deoxyuridine) is a modified thymidine analogue incorporated into DNA during the S-phase of the cell cycle and was used to assess proliferative activity. Coverslips containing cells were fixed in 4% paraformaldehyde for 15 min at room temperature. The cells were then permeabilized in cold methanol for 10 min at room temperature, followed by 1× PBS before proceeding with the immunofluorescence protocol.

For labeling studies using coverslips for EdU, the samples were processed as per the manufacturer’s instructions prior to immunofluorescence.

Immunofluorescence samples were blocked in 5% bovine serum albumin and Triton X-100 0.03% in 1× PBS, washed 3 times in 1× PBS, and incubated overnight at 4 °C with the following primary antibodies in a blocking solution: mouse anti-Ki67 (Dako, part of Agilent Technologies, 5301 Stevens Creek Blvd, Santa Clara, CA 95051, USA; Cat. # M7240; 1:100) and rabbit anti-pSTAT3(y705) (Cell Signaling technology, 3 Trask Lane, Danvers, MA 01923, USA; #9145S; 1:250). After performing 3 washes with 1× PBS, the samples were incubated with the following secondary antibodies at 37 °C for 2 h: goat anti-mouse IgG Alexa488 (Thermo Fisher Scientific, Cat. # A21131; 1:1000) and goat anti-rabbit IgG Alexa594 (Thermo Fisher Scientific, Cat. # A11012; 1:1000).

Following incubation with secondary antibodies, the samples were washed 3 times with 1× PBS and incubated with 0.5 µg/mL of Hoechst 33,258 for 10 min at room temperature for nuclear counterstaining. The samples were mounted using ProLong Gold (Thermo Fisher Scientific, Cat. # P36934) and imaged and quantified using Cytation 5 (Agilent Technologies, Santa Clara, CA, USA).

### 3.4. Flow Cytometry

After treatment under different conditions, the cells were trypsinized to achieve cell suspension and washed with PBS plus 1% FCS. For immunophenotyping analyses, flow cytometry was performed by staining with anti-human surface APC anti-human CD24 (Biolegend, Cat. # 311118, San Diego, CA, USA), Brilliant violet421 anti-human CD44 (Biolegend, Cat. # 338810), and intracellular antibody mouse PE anti-human Ki-67 (Biolegend, Cat. # 350504) according to the manufactured indications for 30 min on ice in the dark. After this, the cells were washed with PBS plus 1% FCS, fixed with fixation buffer (BD Biosciences, San Jose, CA, USA), and then stored at 4 °C until acquired by FACS Canto II (BD Biosciences, San Jose, CA, USA). At least 20,000 events were acquired for each analysis. The data were analyzed using FCS Express 7 Research Edition software (version 7.0).

### 3.5. Statistical Analysis

Kruskal–Wallis tests were used to assess differences between groups, and Dunn’s test was applied for post hoc analysis. A significance level of *p* < 0.05 was considered statistically significant. In vitro analyses are representative of at least three independent experiments.

## 4. Discussion

Tumor cells resistant to treatment are the source of fatal recurrences in breast cancer patients. While we have identified several mechanisms of resistance currently in clinical validation, we are still unable to predict the response to therapy in most cases. Being able to identify features of poor prognosis and novel targets associated with tumor treatment resistance is key to improving survival outcomes in breast cancer patients.

In this study, we addressed this need by characterizing treatment-resistant cells and evaluating their dependency on survival pathways. We developed an experimental methodology to induce tumor cell dormancy that enabled the characterization of features associated with poor prognosis and recurrence after treatment, such as the emergence of the CSC marker CD44. Additionally, we interrogated the dependency on the JAK1–STAT3 axis in maintaining this tumor-resistant cell identity based on evidence supporting the role of IL-6 signaling in promoting CSC treatment resistance [26]. We showed that HER2 inhibition using lapatinib induces a reversible dormant state characterized by reduced proliferation and a predominance of CD44-positive cells during treatment. These findings highlight the importance of CD44 as a marker of cancer stem cell-like properties and its potential role in tumor dormancy that may lead to therapy resistance and disease recurrence.

Our results indicate that lapatinib treatment induces dormancy in the HER2-amplified breast cancer cell lines BT-474 and SK-BR-3. The significant reduction in cell proliferation markers positivity, as well as in cell viability, after HER-2 inhibition and the subsequent resumption of proliferation upon drug withdrawal underscores the reversible nature of this dormancy state, which is consistent with previous reports on residual tumor cell (RTC) behavior following targeted therapy [5,7,11,33,34,35,36,37]. While additional dormancy markers such as p27^Kip1, NR2F1, or gene expression signatures have been shown to characterize this state; these are often context-specific and may vary by tumor type or experimental model. Nevertheless, reversible quiescence remains a distinct and broad feature of tumor cell dormancy

We observed a significant increase in CD44 expression in response to treatment. Unlike other studies, we observed a rapid induction of CD44 within hours of treatment, preceding measurable effects on cell viability, which suggests that CD44 plays an early and critical role in the cellular response to HER2 inhibition [38,39,40]. Moreover, the predominance of CD24^+^/CD44^+^ and CD24^−^/CD44^+^ populations during lapatinib treatment and their robust proliferative capacity post-treatment highlight the resilience of CD44-expressing cells. While the existing evidence supports the pre-existence of CD44-positive populations positively selected upon neoplastic treatment, our findings also support the scenario where the CD44-positive populations may be rapidly induced in response to therapy.

Our study also suggests differential sensitivity to HER2 inhibition among cellular subpopulations. The CD24^+^/CD44^−^ population demonstrated greater susceptibility to lapatinib, this is consistent with the association between HER2 activity and CD24 expression [34,35]. On the other hand, CD44-positive populations (CD24^+^/CD44^+^ and CD24^−^/CD44^+^) exhibited sustained or enhanced proliferative potential during the recovery phase, highlighting the association between CD44 and cancer stem cell-like traits, including enhanced survival, self-renewal, and therapeutic resistance.

In breast cancer cell lines, CD44 inhibition causes a decreased in CSC characteristics, invasion, and metastatic potential [41,42,43]. Importantly, the IL-6/JAK/STAT3 signaling pathway was shown to be essential for maintaining the stem-like characteristics of CD44-positive cells [44]. Similarly, a previous report showed that constitutive inhibition of PTEN expression in BT474 cells triggers the activation of an IL-6 inflammatory loop via the JAK–STAT3 axis that is critical in mediating treatment resistance in HER2-positive breast cancer by expanding the cancer stem cell population [26].

Our data suggest that the inhibition of JAK1 may reduce the CD44-positive population and impair proliferation during the recovery phase. This supports a potential role for JAK1 in sustaining the CD44-positive phenotype associated with resistance to HER2-targeted therapy, although these findings did not reach statistical significance. In contrast, STAT3 inhibition had no observable effect on CD44-positive populations. These differences may reflect a lesser contribution of the IL-6 inflammatory loop to treatment resistance in the SK-BR-3 model. Additionally, because PTEN expression was not suppressed in our system—as it was in the study by Korkaya et al.—the role of PTEN loss in triggering the IL-6 inflammatory loop may be less pronounced in our model [26]. Nevertheless, the transient effects observed and the absence of statistical significance in some assays highlight the need for further investigation into the contributions of the JAK1–STAT3 axis in therapy resistance.

### 4.1. Implications for Therapy Resistance and Clinical Strategies

The persistence and recovery of CD44-positive populations after HER2 inhibition have important implications for therapeutic strategies. While CD44 is a well-established marker of cancer stem cell features, its direct targeting remains challenging due to the diversity of CD44 isoforms and their context-dependent roles [45,46]. In this study, we used CD44 as a biological readout to investigate the interplay between dormancy and stemness in HER2-amplified breast cancer cells. Our findings suggest that JAK1 signaling may regulate both CD44 expression and dormancy-associated phenotypes, providing a rationale for further exploration of this axis.

Although we did not directly test CD44 inhibition, prior work has shown that disrupting CD44 expression or downstream stemness pathways can impair therapy resistance [24,47,48,49,50,51,52,53]. Targeting JAK1-dependent survival mechanisms that sustain CD44-positive dormant cells could therefore enhance the efficacy of HER2-targeted therapies. This concept is aligned with emerging strategies to eradicate tumor cells resistant to primary therapy—likely in dormancy—to prevent recurrence [54]. Combining HER2 inhibition with approaches that eliminate or disable dormant, stem-like tumor cells may help reduce therapeutic relapse and improve long-term outcomes in HER2-positive breast cancer.

### 4.2. Limitations and Future Directions

While our findings provide valuable insights into the role of CD44 in therapy resistance, some limitations must be addressed. First, this study focused on in vitro models, which may not fully capture the complexity of tumor–microenvironment interactions in vivo. Second, although we used Ki67 and EdU negativity as a marker of reduced proliferation, we acknowledge that tumor dormancy is a complex biological phenomenon that may not be fully captured by cell proliferation markers alone [55]. Therefore, we define dormancy in our model based on functional hallmarks—quiescence and reversibility—supported by both Ki67 and EdU observations.

In conclusion, our study identified CD44 as a critical player in the survival and recovery of dormant tumor cells following HER2 inhibition. Targeting CD44-positive populations or their supporting signaling pathways represents a promising strategy to overcome therapy resistance in HER2-positive breast cancer.

## Data Availability

The raw data supporting the conclusions of this article will be made available by the authors on request.
